# Global Oncology Course: Empowering Global Health Students to Address Cancer Burden in Low- and Middle-Income Countries

**DOI:** 10.1200/GO.23.00356

**Published:** 2023-11-16

**Authors:** Oliver Henke, Hans-Friedemann Kinkel, Frank Mockenhaupt, Beate Kampmann

**Affiliations:** ^1^ Section Global Health, Institute for Hygiene and Public Health, University Hospital Bonn, Bonn, Germany; ^2^ Institute for International Health, Charité—Universitätsmedizin Berlin, Berlin, Germany

## Introduction

Cancer is a global health challenge, with significant disparities in its burden and outcomes between high-income countries and low- and middle-income countries (LMICs). Addressing the fast-growing incidences of cancer in LMICs^1^ requires a well-trained workforce equipped with the knowledge and skills to tackle the complexities of global oncology. Globally, 60% of the cancer incidence and 70% of the cancer-related mortality occur in LMICs.^1^ Opposite to this, the workforce of cancer specialists in LMICs and health-infrastructure is far away from being adequately equipped to respond to the cancer problem,^2,3^ which will double in numbers until 2040 in the least developed countries.^4^

This article presents the establishment of a course module in Global Oncology within the master's program of International Health at Charité—Universitätsmedizin Berlin. The course provides participants with a comprehensive understanding of the challenges and strategies in addressing cancer in LMICs. Thus, the course should ultimately contribute to the global fight against cancer and implement global oncology in academic teaching.

Global oncology is considered an opportunity for international collaborations, partnerships, and research, including a possible career for (experienced) clinicians and faculty.^5^ In addition, courses have been designed to teach about clinical global oncology.^6^ However, limited opportunities for students from nonclinical backgrounds are offered to gain a deeper understanding of this topic. And yet, it is precisely these graduates who will shape the future (global) health policies, and they need to understand the impact of cancer on societies.

In this article, we report about the curriculum, design, and impact of the course, providing a blueprint and motivation for other institutions to implement similar programs.

## Course Design and Curriculum

The Global Oncology course module is accredited by tropEd, an international network of institutions for higher education in international/global health.^7^ It consists of 1 week of lectures and 1 week dedicated to a written assignment. The lecturers, including experts from or with long-standing working experience in LMICs, cover various aspects of global oncology. The curriculum includes the topics introduction to clinical oncology and staging, global epidemiology of cancer, cancer registries, barriers to access treatment, universal health coverage and cancer, national cancer control plans, cancer advocacy, pharmaceuticals in LMICs and handling of cytotoxic medications, cervical cancer and women's health, radiotherapy challenges and opportunities, hematologic malignancies in sub-Saharan Africa, global surgery, palliative care in LMICs, and ethics in global health.

This curriculum first delivers a broad understanding of oncology as a clinical discipline to create an understanding of basic requirements for cancer care among nonmedical participants. Second, the challenges of cancer care in resource-constrained settings with all its facets are highlighted, and finally, solutions and practical experiences are presented.

## Pedagogical Approach

The course combines face-to-face learning and group work, fostering interactive discussions and knowledge sharing among participants with medical and nonmedical backgrounds. Because of the COVID-19 pandemic, the course was initially conducted fully online in 2020 and 2021. In subsequent years (2022 and 2023), it transitioned to an on-site format in Berlin, with interactive online lectures for teachers lecturing from other continents. This blended approach allows participants to engage with experts and peers, enhancing their learning experience.

## Written Assignment and Practical Application

After an overview of global oncology, participants are assigned a task to focus on a specific LMIC. They analyze the country's cancer epidemiology, critically assess available data, and examine the country's response to cancer. The participants then identify gaps in the country's cancer response and propose two impactful interventions to lower the burden of cancer. This assignment encourages participants to think critically, apply their knowledge, and develop practical solutions to address the challenges faced by LMICs. These skills will likely contribute to more implementation research in the future as demanded by Pramesh et al^8^ in Nature Medicine last year.

## Outcomes and Impact

Since its inception in 2020, the Global Oncology course has attracted 40 postgraduate master students from diverse backgrounds. Many participants have chosen topics related to global oncology for their master's theses, and some have pursued careers in the field of cancer after obtaining their master's degrees. The course has served as a catalyst for further research and career opportunities in global oncology.

## Unique Contribution and Student Appreciation

The Global Oncology course module which is offered annually is currently the only one within the European tropEd network^7^ that focuses specifically on oncology in LMICs. Participants appreciate the course's unique opportunity to learn about this pressing health issue, which is often neglected in global health education in Europe and elsewhere. The course design ensures that participants not only understand the magnitude of the problem and its challenges but also learn about effective strategies to tackle cancer diseases on regional, national, and international levels, within the health care system and on political and societal fronts. Alumni of the course will contribute to what Horton^9^ has referred to as upgrade our vision for universal health coverage by including specialist care into health for all.

In conclusion, the establishment of a dedicated course module in Global Oncology at Charité—Universitätsmedizin Berlin has provided students with a comprehensive understanding of the complexities and challenges associated with cancer in LMICs. Through lectures, group work, and written assignments, participants gain valuable knowledge and skills to address the pressing issue of cancer in LMICs. The course has received positive feedback, inspiring participants to pursue further research and careers in the field of global oncology also outside the clinical field. As the only course of its kind within the tropEd network for education in international health, it contributes significantly to filling the gap in global health education concerning cancer in LMICs. Continued efforts to expand such initiatives will help create a workforce and future health policy makers better equipped to address the global oncology challenge and reduce the burden of cancer in LMICs.

## References

[1] International Agency for Research on Cancer (IARC): Cancer Today. Globocan 2020. Lyon, France, IARC, 2021

[2] Fadelu T, Shulman LN: Health policy: Towards greater equity in the global oncology workforce. Nat Rev Clin Oncol 15:270-272, 2018 29485133 10.1038/nrclinonc.2018.31

[3] Mathew A: Global survey of clinical oncology workforce. J Glob Oncol 4:1-12, 2018 10.1200/JGO.17.00188 PMC6223442 30241241

[4] International Agency for Research on Cancer (IARC): Cancer Tomorrow. Globocan 2020. Lyon, France, IARC, 2021

[5] Balogun OD, Choi AMK, Formenti SC: Shaping the path for a global oncology academic career. JAMA Oncol 5:931-932, 2019 31046103 10.1001/jamaoncol.2019.0555

[6] Forbes V, Buteau AC, Hoxha I, et al: Global oncology and cancer disparities education. J Clin Oncol 40, 2022 (16 suppl; abstr 11006)

[7] TropEd: TropEd Network for Education in International Health. https://troped.org/

[8] Pramesh CS, Badwe RA, Bhoo-Pathy N, et al: Priorities for cancer research in low- and middle-income countries: A global perspective. Nat Med 28:649-657, 2022 35440716 10.1038/s41591-022-01738-x PMC9108683

[9] Horton R: Offline: Primary healthcare is not enough. Lancet 402:760, 2023 37659771 10.1016/S0140-6736(23)01843-3

